# Chronological trends in maximum and minimum water flows of the Teesta River, Bangladesh, and its implications

**DOI:** 10.4102/jamba.v9i1.373

**Published:** 2017-03-30

**Authors:** Md. Sanaul H. Mondal, Md. Serajul Islam

**Affiliations:** 1 Department of Social Relations, East West University, Bangladesh; 2 Department of Geography and Environment, University of Dhaka, Bangladesh

## Abstract

Bangladesh shares a common border with India in the west, north and east and with Myanmar in the southeast. These borders cut across 57 rivers that discharge through Bangladesh into the Bay of Bengal in the south. The upstream courses of these rivers traverse India, China, Nepal and Bhutan. Transboundary flows are the important sources of water resources in Bangladesh. Among the 57 transboundary rivers, the Teesta is the fourth major river in Bangladesh after the Ganges, the Brahmaputra and the Meghna and Bangladesh occupies about 2071 km^2^. The Teesta River floodplain in Bangladesh accounts for 14% of the total cropped area and 9.15 million people of the country. The objective of this study was to investigate trends in both maximum and minimum water flow at Kaunia and Dalia stations for the Teesta River and the coping strategies developed by the communities to adjust with uncertain flood situations. The flow characteristics of the Teesta were analysed by calculating monthly maximum and minimum water levels and discharges from 1985 to 2006. Discharge of the Teesta over the last 22 years has been decreasing. Extreme low-flow conditions were likely to occur more frequently after the implementation of the Gozoldoba Barrage by India. However, a very sharp decrease in peak flows was also observed albeit unexpected high discharge in 1988, 1989, 1991, 1997, 1999 and 2004 with some in between April and October. Onrush of water causes frequent flash floods, whereas decreasing flow leaves the areas dependent on the Teesta vulnerable to droughts. Both these extreme situations had a negative impact on the lives and livelihoods of people dependent on the Teesta. Over the years, people have developed several risk mitigation strategies to adjust with both natural and anthropogenic flood situations. This article proposed the concept of ‘MAXIN (maximum and minimum) flows’ for river water justice for riparian land.

## Introduction

The Teesta River is the fourth major transboundary river of Bangladesh, located in the northwest region of the country. Actually, it originates in Chitamu Lake in the Sikkim Himalayas at an altitude of about 7200 m and comes down first to the Darjeeling plain and then to the Duars plain of West Bengal in India (Banglapedia 2014). It enters Bangladesh at the Kharibari border of Nilphamari district (Banglapedia 2014). The Teesta is a perennial, rain- and snow-fed river (Prasai & Surie 2013). The discharge of water through the Teesta varies significantly between dry and wet spells. Historically, the Teesta River has had an average maximum flow of 280 000 cusec and a minimum of 10 000 cusec at Dalia, upstream of the Teesta Barrage in Bangladesh (Khalid 2013). Presently, the dry flow of the river is highly controlled for hydro and for irrigation projects both in India and in Bangladesh (Haque et al. 2014). Excessive control over the Teesta flow has made this mighty river sluggish, and this flow has come down to about 1000 cusec and even 500 cusec during droughts (Khalid 2013) in Bangladesh. Being the lower riparian country in the Ganges–Brahmaputra–Meghna (GBM) river system, Bangladesh faces the problem of uncertainty regarding the quantum of water flowing down from the upstream areas (Rasheed 2008). As the flow of water in the Teesta is controlled by the construction of dams, barrages and other irrigation infrastructure, it has reduced the flow of water for most parts of the north Bengal of Bangladesh (Haque et al. 2014).

The Teesta is a major transboundary river that travels through highlands in Sikkim, the hills of West Bengal and the floodplains of West Bengal (India) and Bangladesh. The river has a length of 414 km, drains an area of around 12 159 km^2^ (Alford 1992) and traverses two different countries – India (83%) and Bangladesh (17%). In Bangladesh, the Teesta River is located in the extreme northern zone and has a steeper gradient of 1 in 2000. As a result, the Teesta is flashy in nature. The northern region of Bangladesh has poor rainfall compared with other parts of the country. The mean annual rainfall is 1971 mm in the northwest, while it is 2300 mm in the rest of the country (Strategic Foresight Group 2013). The Rangpur station has recorded a mean annual rainfall of 2270 mm, of which 1294 mm takes place from June to August (Strategic Foresight Group 2013). The mean daily maximum temperature varies from 20 °C in January to 29 °C in April and June; and the mean daily minimum temperatures range from 9 °C in January and February to 19 °C in July, August and October (Strategic Foresight Group 2013). The major discharge measuring station of the Teesta River is in Kaunia and Dalia (Bangladesh). Bangladesh Water Development Board (BWDB) has engaged in recording and documenting chronological hydrological data of these stations. The significance of the flow and seasonal variation of this river is felt during the lean season when the flow has reduced to below the environmental flow requirements of the river. Sharp reduction of water flow during the dry season has reduced the capacity of the river to transport huge sediment loads and so the Teesta river-bed began to rise after each man-made structure built for irrigation purposes both in Bangladesh and in India (Haque et al. 2014). Consequently, the Teesta has lost its original behaviour and volume of discharge. Moreover, the Teesta plays a key role in flushing silt and sediment deposited during the dry season and is a lifeline for irrigation, agriculture, farming, fishing and navigation in the region (Prasai & Surie 2013).

The Teesta is flowing through the five northern districts of Bangladesh (Gaibandha, Kurigram, Lalmonirhat, Nilphamari and Rangpur districts of Rangpur Division), comprising 35 upazilas and 5427 villages, with an estimated population of 9.15 million in 2011 (Prasai & Surie 2013). The population ratio is 70 for Bangladesh and 30 for India (Rashid 2012). Average density of population per square kilometre of this region has increased from 882 in 1991 to 1131 in 2011 (Bangladesh Bureau of Statistics [BBS] 1991–2011). The Teesta floodplain covers around 14% of the total cultivated land of the country. Most of the inhabitants of the Teesta floodplain are engaged in primary economic activities and very few employment opportunities are available in the nonfarm sector. This river is the main source of water in the northern drought-prone region of the country and the livelihoods of millions of people of this region seriously depend on it. Because of its flashy nature, the Teesta River flood causes unexpected severe damage to standing dry-season crops and property. Moreover, water flow of the Teesta decreases sharply during the dry season (October – April). Both situations (flash flood and drought) seriously affect livelihoods of Teesta-dependent communities.

## Literature review

Most of Bangladesh lies within the catchment area of the Ganges, the Brahmaputra, and the Meghna rivers which mainly drain through Bangladesh into the Bay of Bengal (Brammer 2004). Over 220 rivers criss-cross the country having a total length of more than 24 000 km, and they cover about 7% of the national area (Rasheed 2008). The Teesta is an important transboundary river of the northern region of Bangladesh. In the past, a number of researches have been conducted in the Teesta River in Bangladesh. Based on 40 years’ historic flow data of the Teesta River in Bangladesh, Mullick, Babel and Perret (2010) identified the flow characteristics and environmental flow requirements for the Teesta River (Mullick et al. 2010). Their study suggested that a flow of about 90 m^3^/sec – 120 m^3^/sec for the dry season, in particular for January and February, is essentially required for the sustenance of the river itself. However, in the period 2001–2006 (post-barrage-2), mean flow during the dry season (December–March) was 80 m^3^/sec whereas the mean flow in January, February and March flow was only 40 m^3^/sec, 24 m^3^/sec and 57 m^3^/sec, respectively. Afroz Rahman (2013) studied the transboundary river water flows of the Ganges and the Teesta in Bangladesh and found a significant flow reduction after the construction of the Farakka Barrage and the Gozoldoba Barrage, respectively, on the Indian side (Afroz & Rahman 2013). Furthermore, Khan and Hossain (2001) worked on morphological changes because of the construction of the Teesta Barrage in Bangladesh. Their study revealed the changes in the bed level by 2.5 ft in the past 10 years in the extreme most upstream cross-section and degradation in the downstream part and the impact zone of the Teesta Barrage (Khan & Hossain 2001). Gain et al. (2011) used the box plots methods for analysis of stream flow for different seasons and for different time slices of the Brahmaputra River (Gain et al. 2011).

## Objective of the study

The objective of this research was to investigate the trends of both maximum and minimum flow for the Teesta River in Bangladesh and variance in flow pattern because of the withdrawal and sudden release of water by India through the Gozoldoba Barrage and to identify risk-reduction strategies developed by the communities to cope with uncertain flood situations.

## Materials and methods

The chronological records of stages and discharges of Bangladesh’s rivers are scanty. The flow characteristics of the Teesta River were analysed by calculating monthly maximum and minimum water levels and discharges from 1985 to 2006. Historical hydrological data of Kaunia and Dalia stations were collected from BWDB. All these data were then analysed by plotting graph with Microsoft Office Excel package and Statistical Package for the Social Sciences (SPSS) to visualise the temporal changes in monthly maximum and minimum discharge and water level. The variability of maximum and minimum monthly and yearly discharge at Kaunia and Dalia stations was shown through box-and-whisker plots in SPSS environment. The horizontal line across the box stands for the median flow while the bottom and top edges of the box mark the first and third quartiles, respectively. The crosses beyond the whiskers indicate high and low outliers. To identify the risk-reduction strategies, a field survey was conducted along the banks of the Teesta River in Bangladesh. The study was conducted in Kaunia upazila and Gangachara upazila of Rangpur district and Hatibandha and Kaliganj upazila of Lalmonirhat district. The data were collected through 26 structured questionnaires, 3 focus group discussions and 2 in-depth interviews with respondents.

## Results of the study

### Pattern of flow

**FIGURE 1:**
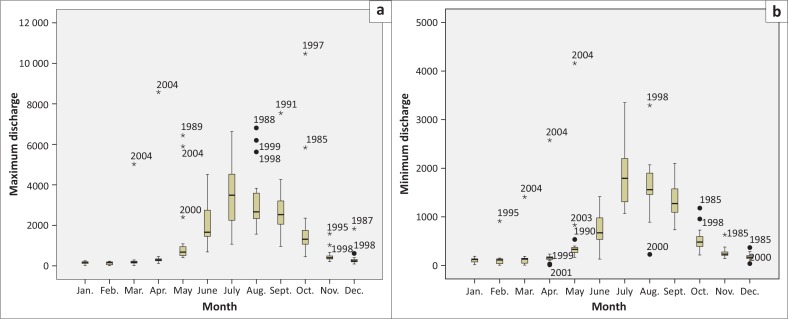
Monthly (a) maximum discharge and (b) minimum discharge at Kaunia from 1985 to 2006.

**FIGURE 2:**
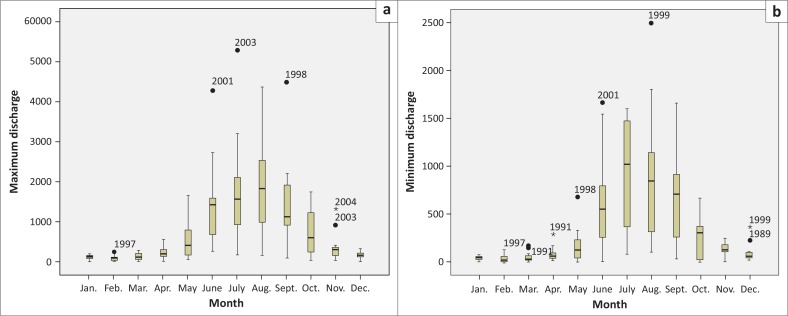
Monthly (a) maximum discharge and (b) minimum discharge at Dalia from 1985 to 2006.

Historical maximum and minimum discharge data of the Teesta River at Kaunia and Dalia stations from 1985 to 2006 were analysed to understand the temporal variation of maximum and minimum flow. Analyses of historical discharge data from 1985 to 2006 at Kaunia station showed that the volume of water rises after March and reaches its peak in April as shown in Figure 1. Afterwards, there was recession of water which continued till June. The discharge of water increased again and hit the highest point in September. The volume of water in January and February was lean. The highest discharge at Kaunia station was 8577 m^3^/sec on 21 April 2004 (Figure 1a) and the lowest flow was 5.47 m^3^/sec on 15 February 2005 (Figure 2b).

On the contrary, at Dalia station, the flow of water increased from mid-May and continued till mid-September with a rapid recession from mid-September and reached the least in January, February and March as shown in Figure 2. The highest discharge at Dalia station was 5294.21 m^3^/sec on 10 July 2003 (Figure 2b) and the lowest flow was 0.05 m^3^/sec on 24 November 1987 (Figure 2b). The maximum discharge from November to April never exceeded 1000 m^3^/sec and the minimum discharge from October to June always hung on below 20 m^3^/sec.

### Yearly recorded maximum and minimum discharge at Kaunia station

**FIGURE 3:**
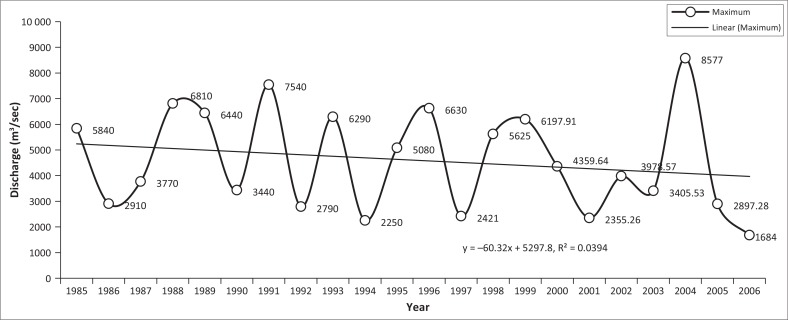
Yearly recorded monthly maximum discharge at Kaunia station from 1985 to 2006.

**FIGURE 4:**
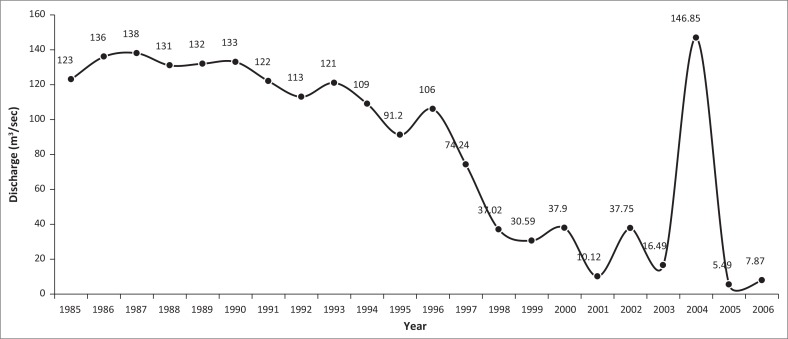
Yearly recorded minimum discharge at Kaunia station from 1985 to 2006.

Irregularities were also observed in the Teesta River flow. The maximum line showed a fluctuating tendency and hit the highest point (8577 m^3^/sec) on 21 April 2004 as shown in Figure 3. The discharge exceeded 6000 m^3^/sec in 1988, 1991 and 1999. Moreover, the yearly minimum discharge graph was quite shocking as discharge had been decreasing year after year, except in 2004. River flow has been regulated since 1987 when India constructed an irrigation barrage at Gozoldoba (Mullick et al. 2010). The biggest criticism is that water from the Gozoldoba Barrage is being diverted to other basins leading to interbasin transfer, leaving very little water to flow down to Bangladesh (Strategic Foresight Group 2013). After the construction of the Gozoldoba Barrage, the flow of water at Kaunia station has been decreasing. The lowest flow of water ever recorded was a mere 5.49 m^3^/sec on 15 February 2005 (Figure 4).

**FIGURE 5:**
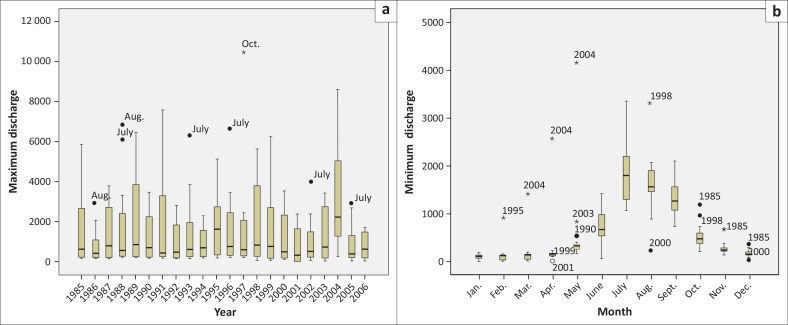
Box plot of year-wise recorded (a) maximum discharge and (b) minimum discharge at Kaunia station from 1985 to 2006.

Minimum monthly discharge at Kaunia station during December to April from 1997 to 2006 never crossed 100 m^3^/sec, while maximum discharge below 50 m^3^/sec was observed during January–March from 1999 to 2006 (except 2000 and 2004) and below 100 m^3^/sec from 1998 to 2006 (except 2004) during December–March (Figure 4). Before the implementation of the barrages, the monthly maximum discharge range fluctuated between 100 m^3^/sec and 7000 m^3^/sec, but irregularities were observed after 2001. The ranges of monthly maximum discharge (monthly recorded lowest–maximum discharge and the peak discharge, respectively) were 9.8 m^3^/sec (04 February) to 2355.26 m^3^/sec (07 October) in 2001; 37.75 m^3^/sec (10 February) to 3978.57 m^3^/sec (28 July) in 2002; 16.49 m^3^/sec (23 February) to 3405.53 m^3^/sec (13 July) in 2003; 25.76 m^3^/sec (03 April) to 8577.00 m^3^/sec (21 April) in 2004; 5.49 m^3^/sec (15 February) to 2897.28 m^3^/sec (17 July) in 2005; and 7.87 m^3^/sec (09 March) to 1684 m^3^/sec (25 September) in 2006 as illustrated in Figure 5. On the contrary, in 1985 (pre-Gozoldoba), the range of monthly maximum discharge was from 123.00 m^3^/sec (25 February) to 5840 m^3^/sec (19 October); 122.00 m^3^/sec (19 January) to 7540 m^3^/sec (11 September) in 1991; 91.20 m^3^/sec (07 February) to 5080.00 m^3^/sec (08 July) in 1995 and 37.96 m^3^/sec (31 December) to 4359.64 m^3^/sec (10 September) in 2000.

Year-wise maximum and minimum discharge from the Teesta River at Kaunia station is shown in Figure 5. The mean maximum and minimum discharges at Kaunia station were 1424.64 m^3^/sec and 643.25 m^3^/sec, respectively, from 1985 to 2006. During the same period, maximum and minimum median discharges were 613.59 m^3^/sec and 291.05 m^3^/sec, respectively.

### Yearly recorded maximum and minimum discharge at Dalia station

**FIGURE 6:**
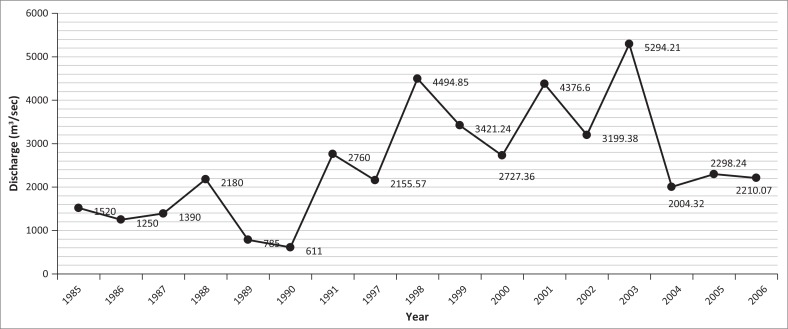
Yearly recorded maximum discharge at Dalia Station from 1985 to 2006.

**FIGURE 7:**
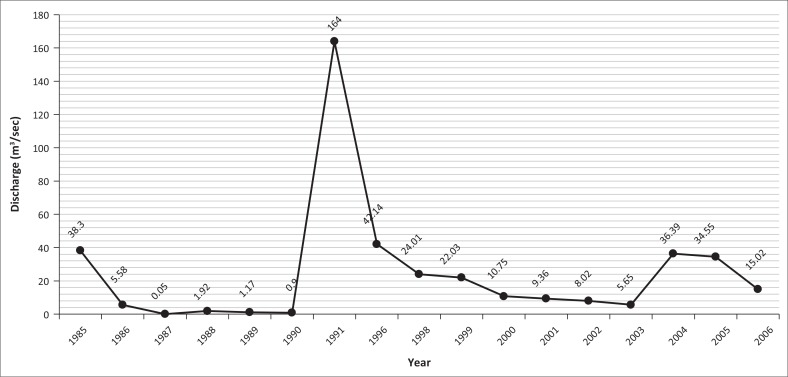
Yearly recorded minimum discharge at Dalia station from 1985 to 2006.

Bangladesh established a barrage on the Teesta at Dalia-Doani point in Lalmonirhat district 1990. From the records available since 1985, Dalia station had its minimum flow of 0.05 m^3^/sec on 24 November 1987. On the contrary, the discharge at Dalia station attained its peak of 5294.21 m^3^/sec on 10 July 2003 (Figure 6). The maximum discharge graph was fluctuating in nature and did not show any smoothing behaviour. Besides, the minimum flow graph was always below 60 m^3^/sec except in 1991 (Figure 7).

**FIGURE 8:**
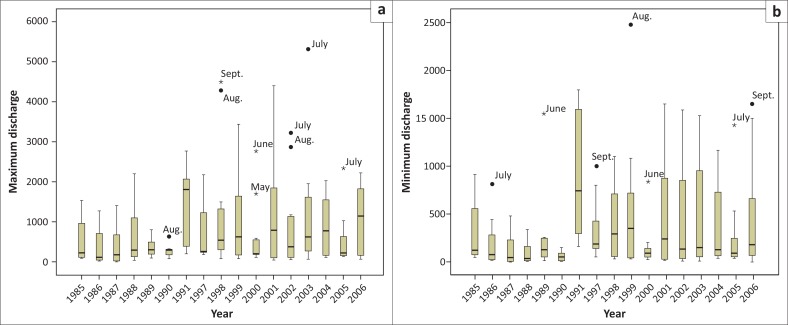
Box plot of year-wise recorded (a) maximum and (b) minimum discharge at Dalia station from 1985 to 2006.

During the period 1985–2006, mean maximum and minimum discharges were 782.61 m^3^/sec and 343.02 m^3^/sec, respectively, as shown in Figure 8. During the same period, median maximum and minimum discharges were 296.64 m^3^/sec and 111.82 m^3^/sec, respectively (Figure 8).

### Variations in monthly maximum and minimum water levels

With the increase in the discharge of the rivers, it is only normal that water levels will also rise, although the highest discharge and the highest stage may not always be coincident in date, because of various factors (river width, river depth, topography, velocity of flow, sedimentation behaviour of river and so on) that interfere with the behaviour of rivers. However, the general pattern of rise and fall of water discharges and those of water levels closely resemble each other. The general picture of the changing level of water over some years is described herein.

### Monthly recorded maximum and minimum water level at Kaunia station

**FIGURE 9:**
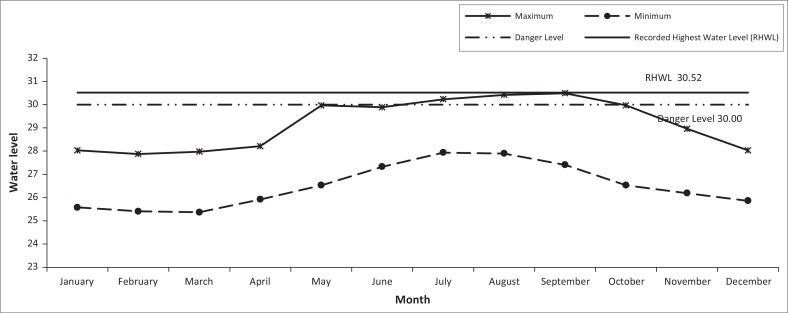
Monthly recorded maximum and minimum water level at Kaunia station from 1985 to 2006.

The monthly maximum and minimum water levels at Kaunia station from November to March (1985–2006) never crossed the danger level as observed from Figure 9. The maximum graph depicted a remarkable scenario. It showed that the water level increased gradually after March and touched or crossed the danger level between May and September. Afterwards, the water level decreased. From the records available, in September the stage reaches its peak level. The highest stage recorded at Kaunia was 30.52 m, PWD (Public Water Datum), on 06 January 1968 (BWDB 2014).

### Yearly recorded maximum and minimum water levels at Kaunia station

**FIGURE 10:**
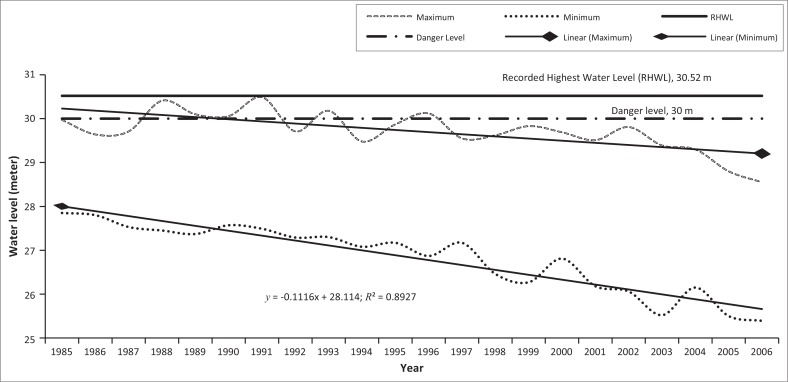
Yearly recorded maximum and minimum water level at Kaunia Station from 1985 to 2006.

From the recorded data since 1985–2006 at the Kaunia station, it was observed that the maximum stage graph line showed a fluctuating tendency between 1985 and 1996 and within this period, the water level crossed the linear line six times (Figure 10). After 1996, the water level never crossed the danger level. The analysis also shows that after 1996, the water level decreased year after year and in 2006, the maximum water level recorded was 25.39 m, which was the lowest ever.

### Monthly recorded maximum and minimum water level at Dalia station

**FIGURE 11:**
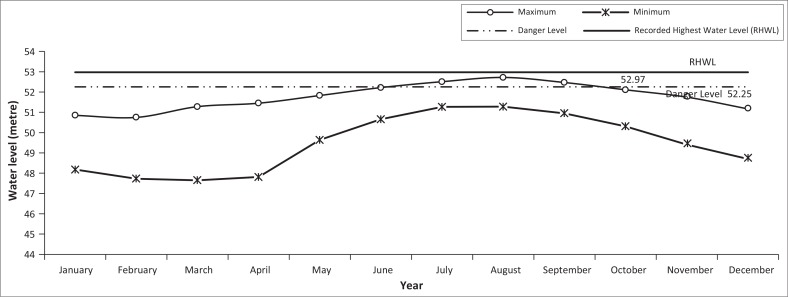
Monthly recorded maximum and minimum water level at Dalia station from 1985 to 2006.

The stage of water at Dalia station increased from April and continued till August, after which the water level decreased as found in Figure 11. The minimum line was just like a parabola in shape whereas the maximum line also exhibited such tendency (Figure 11).

### Yearly recorded maximum and minimum water level at Dalia station

**FIGURE 12:**
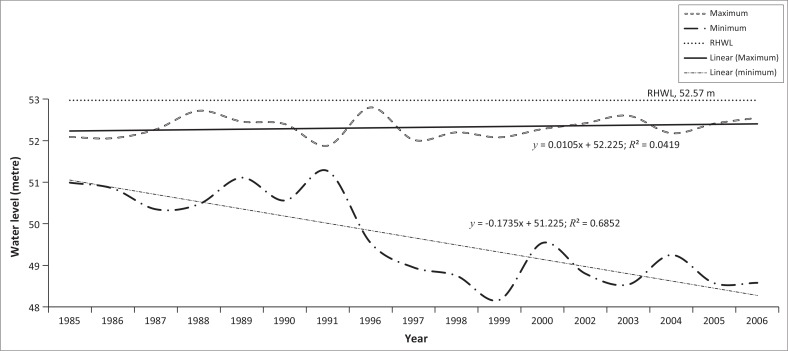
Yearly recorded minimum water level at Dalia station from 1985 to 2006.

Since 1985, the water level at Dalia station exceeded danger level 10 times (Figure 12). The highest stage at Dalia was recorded at 52.97 m, PWD on 29 July 1972 (BWDB 2014).

### Changes in danger level at Dalia Station

The danger level of the Teesta River at Dalia station was changed because of the excessive sedimentation on the riverbed. One of the strong arguments for this excessive sedimentation was the construction of two barrages over the Teesta River–Gozoldoba Barrage (India) as well as the Teesta Barrage (Bangladesh). The Teesta is mighty and flashy in nature and India withdraws Teesta water during the dry season through Gozoldoba Barrage. Excessive control of water at Gozoldoba made the Teesta sluggish during the dry season. As a result, massive sedimentation occurred each year which in turn resulted in the changes of danger level from 52.15 m PWD (up to 2007) to 52.4 m PWD (in 2008) by the BWDB. This indicated the aggradation of sediments because of the construction of the barrages.

## Risk-reduction strategies developed by the communities

**FIGURE 13:**
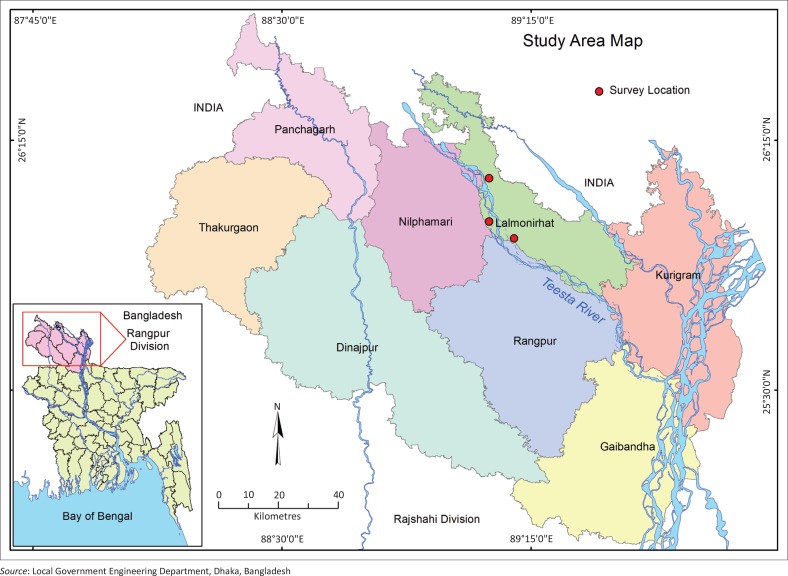
Map of the study area.

The Teesta River flow is very crucial for Bangladesh. It determines the fortune of Teesta-dependent inhabitants. Upstream diversion of water (from Gozoldoba point) during the dry season causes water scarcity, droughts and seriously hampers irrigation; sudden release of water during the rainy season causes floods, river bank erosion and damage to crops in the northern part of Bangladesh. This research found drastic reduction in flow in the dry season from 1985 to 2006. Reduction in dry season flow has seriously been affecting irrigation, during the *Boro* season (dry season); navigation; fishery; and ecology of northern districts (location of study areas shown in Figure 13). On the contrary, sudden release of water from the Gozoldoba Barrage resulted in unusual flood in the northern zone. This uncertain anthropogenic flood risk left Teesta-dependent people more vulnerable. This section is an effort to identify the flood-risk-reduction strategies developed by the communities over the last three decades. Data for understanding risk-reduction strategies developed by the communities were collected from the elderly (aged ≥ 50 years) who resided along the banks of the Teesta River in Bangladesh. The respondents stayed in *Katcha* houses (straw, bamboo or mud-made houses), susceptible to floodwater.

In general, the Teesta River is mighty and flashy in nature. People who are residing along the banks of this river generated coping strategies to adjust to its behaviour. But unpredictable flow pattern over the last two decades have left the inhabitants in a more risky situation than the pre-Gozoldoba Barrage period. Almost all the respondents fall within the low-income category with monthly income less than BDT 4000 (1 USD = BDT 80). However, one-fourth of the respondents worked as *Adhiars* (the sharecropper) for the landowners.

All the respondents identified the Teesta River flood as both natural (overflow of river banks) and human-made (sudden release of water from the upstream). Actually, sudden release of huge volumes of water from the reservoir was the main reason for uncertain floods in their locality in recent decades. Every respondent had experienced floods almost every year in their lifetime but the severity and extension of floods varied with respondents and location of houses from the river. To discover the coping strategies and livelihood measures in a flood, respondents were asked about the duration and extension of flood stage. Actually, topography determines the duration and extension of flood level in the study area. People from *Char* areas (A *Char* land is a tract of land surrounded by the waters of an ocean, sea, lake, or stream. source: Banglapedia) experienced sudden rise and rapid recession of water level, whereas in other areas (plain land), floodwater stayed for more than 11 days as identified by the survey. Because of its low topography, *Char* areas are easily overflowed by floodwater and it is hard to find unsubmerged places during flooding stage.

Uncertain floods did cause immense damage to the lives and livelihoods of the inhabitants. *Katcha* houses are more susceptible to moderate wind and even water as these are made of locally available bamboo, straw and jute sticks. As a result, every year these houses were damaged easily in the monsoons or rotted during the floods. All of the respondents relocated their houses more than three times in their lifetime as their houses were affected by flood or flood-induced riverbank erosion. However, they had to spend some money (average USD 70.00 per household) to rebuild or reconstruct their houses every year. Hence, the risk factor because of floods in terms of monetary value was also high. Flood is also linked to an increased risk of diseases and infections. Almost all the respondents suffered from diarrhoea, dysentery, scabies, fever and conjunctivitis during and after floods. Access to safe drinking water was a crucial issue for all respondents.

To cope with these adverse uncertain floods situation, people developed some risk-reduction strategies that are discussed in the flowing sections.

### Shelter during floods

All the respondents had to leave their houses during floods as their houses were submerged in flood water. They took shelter on embankments, roads or nearby schools. Respondents hardly took refuge in the flood shelter centres for various reasons such as flood shelter centres being located in distant places, no separate place for women, inadequate facilities among others.

### Source of help in case of flood condition

Flood insurance is not available in Bangladesh and usually people are forced to seek immediate help from sources available to them during or after the occurrence of floods. Almost all the respondents hardly get external support in such a situation. Majority of the respondents relied on their relatives for help during a crisis. Sources of help from local administration or government and non-government organisations (NGOs) were too meagre. It was interesting that most of the respondents depended on their own resources or savings.

### Agricultural rehabilitation after flood phase

The post-flood agricultural rehabilitation was very crucial for the respondents. As soon as the floodwater starts receding, respondents who have family connections on high land look for seedbed there, if available, for sowing seeds. Respondents also searched for assistance or loans from NGOs for cultivation. Almost half of the respondents took loans from the *Mohanjon* (local money lender) with a high interest rate (interest rate varies from 15% to 25% on principal amount per month). They used the money for purchasing domestic animals, fertiliser, seed and other agricultural equipment.

### Individual-level coping strategies

This research made an interesting finding that at present people are more responsive to floods than earlier. Respondents have taken different short-term and long-term measures to cope with recurrent uncertain floods. Almost every respondent either raised the plinth of their homestead or strengthened their house with strong materials (such as bricks, corrugated iron roofs, woods) or both. Some respondents have started to generate savings at the family level or at local NGOs to face the crisis during or after floods. Around half of the respondents have engaged in backyard poultry rearing to get hard cash during the crisis period. The respondents could sell out their chickens at the onset of floods. It helps them enormously before or after the floods, whereas the ducks sell out at a later period of flood as they can survive even in water. However, some of the respondents are incapable of saving or taking any other necessary steps. But they have knowledge of coping strategies. Respondents (80%) also received capacity development trainings on risk reduction and coping strategies from government and development organisations. These trainings include agricultural risk management (selecting flood- or drought-resistant crop varieties), adjustment of seasonal crop calendars, income-generating activities for women, capacity development of women on flood preparedness at family level (such as food preservation) and so on.

## Discussions and conclusion

The analysis was based on the recorded discharge and water level of the Teesta River at Kaunia (located about 70 km downstream of the Teesta Barrage at Dalia) and Dalia stations. The analyses showed that substantial reduction of maximum and minimum discharge had taken place in both stations, especially in the recent past from the year 1990, that resulted in an alarming situation in the downstream part of the Teesta. Reduction in dry season flow may have significant socioeconomic impacts downstream by altering the hydrological pattern and accelerated sedimentation, which in turn affect livelihoods of local communities. The flow of the Teesta is closely associated with the agriculture and livelihoods of the northern region of Bangladesh. It is evident from the above analysis that the variation of flow within the year was disproportionate; however, considerable amount of flow reduction has taken place, especially in the recent past for dry and wet seasons.

From 1997 to 2006, the maximum water level at Kaunia station never crossed the danger level and the maximum value showed a decreasing tendency. On the contrary, at Dalia station, minimum water level had been decreasing sharply since 1991. The maximum water level was also decreasing with few fluctuations. Probably, these fluctuations result from excessive sedimentation in the riverbed. The Teesta Riverbed is being aggradated at a rate of 100 mm per year upstream of the barrage since its construction (Khan & Hossain 2001). Exclusive control of Teesta’s water in the dry season at Gozoldoba makes the Dalia Barrage useless, and furthermore, the sudden release of excessive water through the Gozoldoba Barrage (India) in the rainy season causes flood and bank erosion, and leads to serious suffering for the people in the Bangladesh area of the basin (Higano & Islam 2002).

The analyses set out above designate that the diversion of water from the Gozoldoba Barrage has caused noteworthy changes in the dry season discharge (November–March) of the Teesta River in Bangladesh. However, the sudden release of excess water from the Gozoldoba Barrage caused severe losses to the people. Thus, reduced flow in the dry season and the sudden release of water has potentially wide-ranging socioeconomic and environmental implications for Bangladesh. Because the Teesta is a transboundary river between India and Bangladesh, water sharing is a crucial issue.

Recurrence of floods in the Teesta River is no mean problem for the respondents. They perceived floods as both a natural and human-made phenomena, but in many cases they believed flood to be the ‘will of God’. Therefore, they are not afraid of floods as this is a common and regular phenomenon almost every year in their locality. But respondents are scared of uncertain huge flows from the Teesta released from the Gozoldoba Barrage.

Results from this research will provide basic but crucial information to the researchers and policy makers for effective and sustainable management of the Teesta River catchment in lower riparian-Bangladesh. In conclusion, it is to be said that this research might act as a helping tool while discussing Teesta water sharing issues by introducing a new concept of ‘MAXIN (maximum and minimum) river water justice for riparian’. The gross discharge over a year through a river may be the same, but it is a serious issue as to whether this water is equally distributed over the year or not. Thus, a legally binding agreement should be executed wherein India should discuss with Bangladesh before releasing huge amounts of water owing to unexpected melting of snow or excessive rainfall in the upper catchment area. In addition, the gap between maximum and minimum discharge through the Teesta should be as low as possible to maintain the ecological health of the river and prioritise the intrinsic value of the river.
